# Headline indicators for monitoring the performance of health systems: findings from the european Health Systems_Indicator (euHS_I) survey

**DOI:** 10.1186/s13690-018-0278-0

**Published:** 2018-06-28

**Authors:** Nataša Perić, Maria M. Hofmarcher, Judit Simon

**Affiliations:** 1 Department of Health Economics, Centre for Public Health, Medical University of Vienna, Kinderspitalgasse 15/1, 1090 Wien, Austria; 2 HealthSystemIntelligence, Josefstädterstraße 14, 1080 Wien, Austria

**Keywords:** Health system performance assessment, Indicators, Health information, Policy making, International comparisons, European Union

## Abstract

**Background:**

Cross-country comparisons of health system performance have become increasingly important. Clear evidence is needed on the prioritization of health system performance assessment (HSPA) indicators. Selected “leading” or “headline” HSPA indicators may provide early warnings of policy impacts. The goal of this paper is to propose a set of headline indicators to frame and describe health system performance.

**Methods:**

We identified overlaps and gaps in the availability of reported indicators by looking at HSPA initiatives in Member States (MSs) of the European Union (EU), the European Commission as well as international institutions (e.g. OECD, WHO-EUR). On that basis, we conducted a two-stage online survey, the european Health System_Indicator (euHS_I) survey. The survey sought to elicit preferences from a wide range of HSPA experts on i) the most relevant HSPA domain(s), i.e. access, efficiency, quality of care, equity, for a specific indicator, and ii) the importance of indicators regarding their information content, i.e. headline, operational, explanatory. Frequency analysis was performed.

**Results:**

We identified 2168 health and health system indicators listed in 43 relevant initiatives. After adjusting for overlaps, a total of 361 indicators were assessed by 28 experts in the 1st stage of the survey. In the 2nd stage, a more balanced set of 95 indicators was constructed and assessed by 72 experts from 22 EU MSs and 3 non-EU countries. In the domain *access* experts assessed share of population covered by health insurance as the top headline indicator. In the domain *efficiency,* the highest rank was given to Total health care expenditure by all financing agents, and in the domain *quality of care* to rate of hospital-acquired infections. Percentage of households experiencing high levels/catastrophic of out-of-pocket health expenditures results as the top headline indicator for domain *equity*.

**Conclusions:**

HSPA indicators from different initiatives largely overlap and public health indicators dominate over health systems aspects. The survey allowed to quantify overlaps and gaps in HSPA indicators, their expert allocation to domain areas and establishment of an informed hierarchy structure. Yet, results show that more multidisciplinary work is needed to ensure the availability of accurate efficiency indicators which are comparable across countries.

**Electronic supplementary material:**

The online version of this article (10.1186/s13690-018-0278-0) contains supplementary material, which is available to authorized users.

## Background

Health system performance assessment (HSPA) is a topical issue. The World Health Organization (WHO) describes HSPA as “a country-owned process that allows the health system to be assessed holistically, a ‘health check’ of the entire health system” [1]. HSPA has now received high-level support at national, European Union (EU) and broader international (WHO, Organisation for Economic Co-operation and Development - OECD) levels as an instrument to improve transparency and accountability [2, 3]. For example, the European Commission’s (EC) communication on effective, accessible and resilient health systems [4] and the mandate by the EC President given to the European Commissioner for Health to develop expertise for HSPA reflect this [5].

While policy making in many areas of EU health systems is the responsibility of Member States (MSs), comparisons of health system performance (HSP) have become increasingly important to foster cross-country learning. Therefore, the EC supports MSs directly in this work by providing analysis and forecasts, and recommending reforms based on evidence linked to robust and comparable information [6–8]. Reflecting generic policy goals, HSP is measured against multiple objectives. This calls for a strong framework covering access, efficiency, equity and quality and their interrelation in order to understand the content and the scope of the cross-country comparison [9, 10]. In undertaking international comparisons, it is crucial to have good understanding about the strengths and limitations of existing indicators, and their usefulness in assessing system performance [11, 12].^.^

Although notable achievements have been made in terms of scope, nature and timeliness of performance data over the last 30 years, methodological challenges remain. In particular, a European-wide coherent HSPA framework for cross-country comparison does not exist [9, 10]. Even though the European Core Health Indicators (ECHI) initiative is an important source of relevant indicators, creating and unifying reporting standards of data and indicators [13] with priority information content is missing [14]. Currently, health and health system indicators for cross-country comparison exist in repositories such as ECHI/Eurostat, OECD health statistics and WHO European health information gateway [15]. A proliferation of HSP indicators at the international level has evolved for a variety of purposes, including informing policy development, evaluating policy initiatives, promoting accountability to citizens, managerial control, and research. This can cause both confusion and duplication of effort, and also leads to a lack of comparability over time and between countries. Both consequences suggest a need to rationalize the collection and dissemination of indicators if their usefulness and impact is to be maximized. Having a manageable set of “leading” or “headline” indicators may provide a focused system overview at a glance. If aligned to health (system) strategy goals or a common framework with a proper definition, they can give early warnings of policy impacts, highlight trends, indicate priorities for policy action, and promote accountability. Ideally, they also foster cross-country learning through stimulating further analysis [16]. This was already advocated by the independent and multidisciplinary Expert Panel on Effective Ways of Investing in Health (EXPH) [13].

The concept of headline indicators as an important monitoring tool to track and explain progress toward strategic targets is well established and has been adopted in various areas, for example, in the Europe 2020′ strategy [17] and in the area of Sustainable Development [18]. It is convention in macro-economics where core indicators of Gross Domestic Product growth, inflation, unemployment, and current account are standard in looking at the performance of countries [19]. Also, the scoreboard of key employment and social indicators echoes the importance of such concepts [20]. For the current study, we have adapted these existing concepts [18, 21] and defined *headline indicators* of health systems as being apt to monitor the overall performance in defined domains related to key objectives in public health and in health systems. Other criteria include being robust, widely used with high communicative and educational values, and available for most EU MSs, generally for a minimum period of 5 years.

The aim of the paper is to identify overlaps and gaps in the availability of used and proposed HSP indicators, and ultimately to provide a set of headline indicators for HSPA. For this, we conducted a two-stage online survey and asked experts’ to map existing indicators onto most appropriate HSPA domain(s) and assess their priority information content for HSPA. The present work forms an integral part of the activities of the team working on the evaluation of health systems within the BRIDGE Health project (Work Package 12). It draws on previous research conducted in the FP7 project EuroREACH [10] and on our earlier paper looking at major HSPA actors and initiatives at European level [15]. To ensure consistency with past and existing initiatives and to inform the survey design, we established the BRIDGE Health System Indicator Task Force [22], a body of high level international experts in the area of HSPA.

The paper is intended both for researchers, as well as for decision makers and policy advisors at EU and MS level by summarizing the key findings in terms of the resulting indicator inventory and the identified “headline” indicators which may be used to frame and describe the performance of a health system across EU countries. The rest of the paper is organised as follows: the Methods section provides details of the applied methods; in Results we report the main findings followed by their Discussion and some main Conclusions.

## Methods

We comprehensively and systematically reviewed, assessed and organized the existing health and HSP indicator landscape for the EU context using a multi-layer approach.

### Inventory of indicators

Firstly, we compiled an inventory by identifying and including reported indicators in i) HSP initiatives at the EU, OECD and WHO-EUR levels [15], ii) similar initiatives developed at the level of MSs when available in English and iii) performance work done in Australia, Canada, New Zealand and US, countries with longstanding experience in the field of HSPA. Based on a previous focused search conducted by the authors between October 2015 and June 2016 that informed a mapping exercise of the HSPA landscape at EU level [15], relevant HSP initiatives at the EU, OECD and WHO-EUR levels were identified. This search was extended to identify relevant initiatives at MS level where mapping reports by the EG HSPA on quality of care [23], country comments of the HSPA Belgium peer-review process [24] and the health system accountability multi-country study by WHO-EUR [25] proved useful primary sources to grey (i.e. institutional websites, reports from national organisations) and peer-reviewed literature (e.g. scientific articles describing development of country HSPA process). For complementing the inventory with an international perspective, also five institutional websites of Australian, Canadian and New Zealand ministries of health and the Commonwealth Fund were searched. We extracted indicators from initiatives that fulfilled the criterion of informing a blueprint for an indicator repository of a European health information infrastructure [15].

Indicator names and all reported corresponding meta-information such as definition, calculation, rationale, and data availability were extracted into an indicator inventory. The inventory was then organized into a total of 20 thematic chapters in line with the chapter structures of the OECD Health at a Glance report from 2015 [26] and the OECD Health Care Quality Indicators (HCQI) Framework [23, 27]. Overlapping indicators were consolidated and in some instances (re)classified. The consolidation was not done through a formal statistical method but by using systematic rules, e.g. eliminate duplicates with similar definitions or disaggregation levels and create respective ‘indicator groups/themes’ of similar indicators with the same denominator but different numerator (e.g. health care expenditures by financing agent, hospital discharges by disease, cancer screening rates etc.). In addition, we used rules such as “rather country-specific and/or lack of information/definition”, and “not relevant and/or out of scope” to consolidate and eliminate further indicators. All steps were done through structured discussions involving the core research team and other WP12 partners with expertise in economics of health, public health, health services research, health policy, and mental health. Identical indicators as well as indicators with similar definitions or disaggregation levels were collapsed. A complete list of what we considered to be rather country-specific indicators is provided in Additional file 1.

### Developing the european Health System_Indicators (euHS_I) survey

Secondly, in order to elicit i) the most appropriate HSPA domain(s) for an indicator, and ii) the indicator’s importance for HSPA based on a pre-defined indicator hierarchy structure, we conducted a two-stage online survey in English.

Two organizing principles informed the vertical and horizontal structure of the euHS_I survey. Reflecting broad health policy goals, the survey used a stylized framework covering access, efficiency and quality as main health system performance domains as well as the cross-cutting domain of equity [28]. Detailed definitions of the key concepts of these broad dimensions are presented in Additional file 2.

For the indicator hierarchy structure, we used the framework developed by the EU Sustainable Development Strategy that proposes a grouping of indicators according to an assessment of their priority information content in the form of an indicator pyramid [18]. A similar three-level approach is used by DG Environment for measuring resource efficiency. The respective indicator set consists of i) one headline indicator, ii) a dashboard of complementary indicators, and iii) a set of theme specific indicators to measure progress towards the specific objectives and actions [21]. We used this approach as it highlights headline indicators which co-exist with larger sets of indicators on operational and explanatory levels for more comprehensive policy-making and monitoring. Also, it avoids creating composite indicators which are often difficult to interpret [29]. However, headline indicators face the limitation that they could be used for politics, rather than policy. Specifically, their choice could reflect current political priorities rather than significant issues influencing future sustainability. Nevertheless, if they are used correctly, they have the potential to attract media attention, raise awareness and more importantly, provide quick and visible signals to policy-makers and to the general public [30].

After pilot testing the content, length, clarity, and ease of use within the HSI Task Force, the 1st stage of the anonymized and revised euHS_I survey was conducted from June to September 2016. This was followed by a 2nd stage from March to May 2017. Our overall participant sampling frame included all EU MSs, the EC and international organisations (OECD, WHO), as well as authors from other included HSPA initiatives from non-EU countries. In the 1st stage, we surveyed a selected number of HSPA experts, i.e. persons actively involved in performance measurement and reporting, indicator development, or research of HSPA domains. We further included partners of the BRIDGE Health consortium, as well as relevant experts from the EC (including the Expert Group on HSPA), the OECD and the WHO-EUR (*n* = 92). In the 2nd stage, a systematic selection of 209 experts from 28 EU MSs, 11 non-EUcountries and two international organisations were asked to complete the survey. Here, the primary aim was to achieve a high and representative response rate from expert representatives of MSs and from international organisations.

Whereas the 1st stage consisted of the full list of the identified consolidated indicators, the 2nd stage was reduced to a more balanced set of indicators prioritised based on the 1st stage results. Prioritisation was done through backward elimination where all indicators that received less than three scores as headline indicator in the 1st stage were excluded (see Fig. 2). While in the 1st stage the level assessment of indicators was a multiple-choice format, it was restricted to single choice in the 2nd stage to enable more conclusive judgment. Every indicator was accompanied by an explanatory information that contained the consolidated definition and a reference list of the source initiative. Furthermore, as a standalone question at the end of the survey, participants were asked to rate the importance of 11 proposed criteria of any headline indicator on a Likert scale of 1–5 (1 = important, 5 = not important), see Table 1. These criteria were derived from a synthesis of applicable information from relevant reviewed initiatives [18, 26, 27, 31–39]. Ultimately, participants were asked to optionally list their top three headline indicators per HSPA domain based on their individual preferences for reasons of cross-validation and allowed to provide comments. Overall, we allowed participants to only assess indicators according to their expertise and made all questions optional to minimize the dropout rate. Participants were contacted by email and two reminders were distributed at earliest two and 5 weeks after the initial invitation.

**Table 1:** Definitions of applied indicator (selection) criteria (*n* = 11)

Criterion	Definition	Source
(policy) Relevance	● The extent to which the measures represent the most critical issues and priorities of the health system*.* ● An indicator measures an aspect of quality with high clinical importance, a high burden of disease or high health care use.	[18, 27, 31, 34, 35, 37, 38]
Actionability	● Monitors the overall performance related to the attainment of key objectives. ● An indicator measures an aspect that is subject to control by providers and/or the health care system and is actually used at a national level for policy making, monitoring or strategy development.	[18, 27, 31–33, 35, 38]
Clear and easy to communicate & interpret	● Indicator is widely used with a high communicative and educational value. ● Measure would be easily understood such that the meaning behind the numbers would be immediately apparent for all stakeholders, from statisticians and measure developers to students, patients, and other individuals.	[18, 26, 31, 32, 34, 37, 38]
Validity	● Sufficient scientific evidence exists to support a link between the value of an indicator and one or more aspects of health care quality.	[18, 27, 31–35, 39]
Reliability	● Repeated measurements of a stable phenomenon get similar results.	[27, 34, 38, 39]
International feasibility	● An indicator can be derived for international comparisons without substantial additional resources.	[18, 27, 34, 38]
International comparability	● Reporting countries comply with the relevant data definition and where differences in the indicator values between countries reflect issues in quality of care rather than differences in data collection methodologies, coding or other non-quality of care reasons. ● It should be possible to compare the indicator over time and ideally between places. ● Comparability is ensured when concepts and definitions follow internationally agreed standards.	[18, 27, 31, 33, 35, 38]
Routine availability	● The indicator should be available for minimum 5 years for most MS.	[18, 26, 31–36, 38, 39]
Far reaching	● A core measure set needs to capture not only progress on the specific measures it includes but also progress on overarching, meaningful priorities for health across the health system, touching on the full range of actors and stakeholders involved and driving improvement throughout	[31, 37]
Coherent and balanced overall	● An indicator set should have an appropriate mix of indicators at different monitoring levels; e.g. there should be indicators to assess inputs, outputs, outcomes and impact.	[34, 35, 38]
Minimum number of indicators	● A core measure set should comprise the minimum number of measures needed to assess health and health care system.	[34, 35, 37]

Frequency analysis was performed. Results in this paper focus on the most frequent “top-level” headline indicators per HSPA domain. For this we gradually calculated three types of frequencies which served as ranking principles to be found in the third column of each table: “HSPA domain frequencies” (Table 4), “headline level frequencies” (Table 5), and “individual preferences frequencies” (Table 6). Based on these frequencies rankings were derived to compile the top three headline indicators per domain. In case of ties in the ranking, all indicators are reported and were given the same rank. Those with the highest ranks among all three were selected. These are accompanied by a summary of provided comments. In addition, we report results on indicator criteria as means with standard deviations and carried out analysis using chi-squared test.

## Results

### Inventory of indicators

**Fig. 1:**
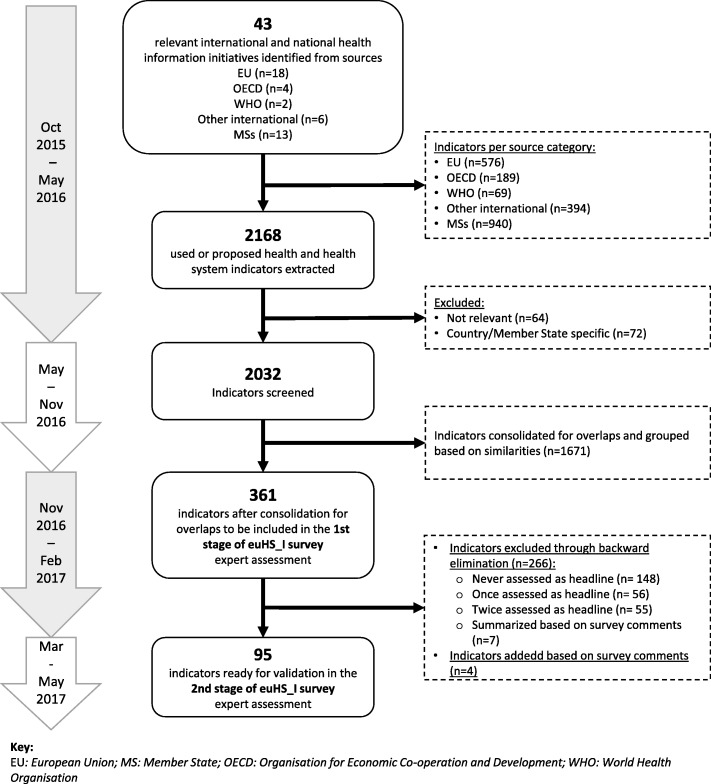
Flow chart with timeline

**Fig. 2:**
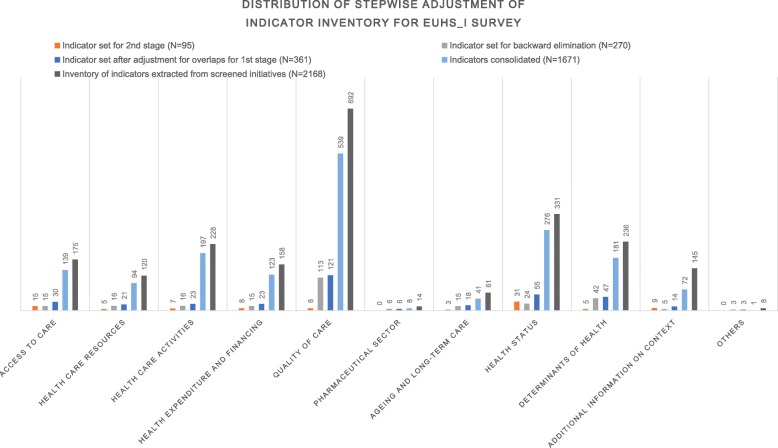
Distribution of stepwise adjustment of indicator inventory for euHS_I survey

Altogether, we included 43 relevant national and international HSPA initiatives coming from the EC [2, 7, 13, 18, 32, 36, 40–55], the OECD [26, 33, 56, 57], the WHO [35, 58], and other international institutions [37, 59–65] and EU MSs [66–78] (Fig. 1). Thirteen initiatives from Belgium, Estonia, Hungary, Ireland, Italy, Malta, Netherlands, Portugal, Sweden and United Kingdom informed the inventory at MS level. The extraction of indicators resulted in a long list of 2168 reported health and HSP indicators, of which 43% were found in reports from MSs. After excluding 132 indicators considered irrelevant for HSPA and/or as being too country-specific and adjusting the remaining 2032 indicators for overlaps, a final list of 361 indicators was included in the 1st stage of the euHS_I survey. Fig. 2 shows the distribution of this initial set of consolidated indicators. We found that indicators listed in the domains quality of care (34%, 121/361), health status (15%, 55/361) and determinants of health (13%, 47/361) dominate the current HSP indicator landscape. In contrast, indicators of financing (23/361), physical resources (21/361) and healthcare activities (23/361) represent only 6% per chapter.

### Characteristics of survey responders

Table 2 describes the characteristics of the euHS_I survey responders by stage. In the 1st stage, we received 28 responses, corresponding to 29% of experts surveyed. Of the total responses, 10 (36%) were complete and 18 (64%) were partially complete through letting participants to focus only on domains that matched their relevant expertise. In the 2nd stage, the overall response rate was 34% (72 out of 209). This increase was mainly achieved through an improved and representative response to the survey coming from EU countries a total of 79% (22 out of 28). In total, out of the 72 responses 52 (72%) were complete and 20 (28%) were partially complete.

**Table 2:** Characteristics of the euHS_I survey responders by stage

Characteristics	1st stage (*n* = 28)		2nd stage (*n* = 72)	
	n	%	n	%
Overall completion rate^a^				
Fully completed	10	36%	52	72%
Partially completed	18	64%	20	28%
Response rate by affiliation				
Governmental or other public institution	14	50%	33	46%
Research institution	12	43%	26	36%
International organization	1	4%s	0	0%
Health care provider	1	4%	10	14%
Non-profit organization	0	0%	3	4%
Geographic distribution by region^b^				
West	13	46%	24	34%
South	7	25%	27	36%
North	5	18%	8	12%
East	2	7%	11	15%
Non-EU	0	0%	2	3%
No response	1	4%	0	0%
Level of HSPA expertise (1 = basic, 5 = expert)				
1 (basic)	1	4%	0	0%
2	5	18%	9	13%
3	3	11%	26	36%
4	7	25%	21	29%
5 (expert)	12	43%	14	19%
No response	0	0%	2	3%
Area of expertise^c^				
Chronic Care	7	25%	9	13%
Determinants Of Health	12	43%	32	43%
Economics	8	29%	6	8%
Elderly / Long-Term Care	2	7%	5	7%
Environmental Health	2	7%	14	19%
Epidemiology	12	43%	37	51%
Ethical Issues	0	0%	6	8%
Financing Health Care	13	46%	11	15%
Health Economics	19	68%	19	26%
Health Spending, Cost of Care	14	50%	9	13%
Maternal And Child Health	4	14%	8	11%
Organization and Delivery of Care	9	32%	15	21%
Pharmaceuticals	2	7%	10	14%
Quality of Care	10	36%	24	33%
Workforce Issues	5	18%	4	6%

^a^Respondents who completed less than 5 indicator were considered as non-responder

^b^According to Eurovoc geographical classification [92]

^c^These categories were not mutually exclusive and hence the sum is greater than 100%

Responders’ affiliation with a governmental or other public institution was 50% (*n* = 14) in the 1st stage and 46% (*n* = 33) in the 2nd stage representing the biggest category in both stages. Participation from research institutions decreased from 43% (*n* = 12) in the 1st stage to 36% (*n* = 26) in the 2nd stage. A considerable level of expertise in HSPA (defined as a score of 3 or higher) of responders was observed in both stages, 79% (*n* = 22) in the 1st stage and 84% (*n* = 61) in the 2nd stage. 68% (*n* = 19) of respondents of the 1st stage were experts in health economics which decreased to 26% (n = 19) in the 2nd stage. The 2nd stage had the highest rate of experts in the area of epidemiology, 51% (*n* = 37) compared to the 1st stage, 43% (n = 12). Overall as shown in Table 3, the mean indicator assessment rate by thematic chapters increased from 58% (209 out of 361) to 72% (69 out of 95) in the 2nd stage.

**Table 3:** Average indicator assessment rate by thematic chapters of both survey stages

Thematic chapters	1st stage		2nd stage	
	n	%^a^	n	%^a^
Access	30	83%	15	92%
Health care resources	21	73%	5	81%
Health care activities	23	68%	7	80%
Health expenditure & financing	23	64%	8	71%
Quality of Care	121	55%	8	71%
Pharmaceutical	6	53%	0	0%
Ageing and LTC	18	52%	3	69%
Health Status	55	56%	32	64%
Determinants of Health	47	46%	5	62%
Additional info	14	49%	12	63%
Other	3	50%	0	0%
TOTAL	361	58%	95	72%

^a^The average number of indicators in percent of the total number of indicators per chapter included in the survey stages. The number of indicators varies because of completion preferences by respondents

### Top three indicators ranked by HSPA domain, headline level and individual preferences

Table 4 lists the most highly ranked HSPA domain indicators which are accompanied by the rank of headline level and the rank it received based on individual preferences. In Table 5 the most important indicators by headline level are summarized. Table 6 presents the ranking of top three listed headline indicators by individual preferences of respondents. Indicators which are marked with a star can be considered as robust “top-level” indicators as their ranks are top, in domain, in headline level, and in individual preferences. If there are duplicate values in the ranking, these are given the same rank. Sample sizes, indicated by N, vary due to differences in completion rates. Tables 5 and 6 further display the availabilities in the most common data repositories. Overall, of those who assessed the respective indicators, on average only 8% of respondents indicated that they do not have appropriate expertise in assessing the relevance and importance of indicators. Explanatory information of presented headline indicators per HSPA domain is available in Additional file 3.

**Table 4:** Top three indicators ranked by HSPA domain frequency

Domain	Name of indicator	Ranking by^a^			Frequencies			
		HSPA domain	Headline	Individual preferences	HSPA domain^b^		Headline^c^	
					N responded	assessed for domain %	N responded	assessed for headline per HSPA domain %
Access	Share of population covered by health insurance	1	1	1	69	96%	43	65%
	Self-reported unmet need for medical care (total by reason: cost, waiting time, distance)	2	3	na	68	91%	25	40%
	Accessibility to acute care	3	2	3	66	92%	26	43%
Efficiency	Average length of stay (ALOS), total and selected diagnoses	1	39	1	58	84%	7	14%
	Hospital beds	2	2	na	61	79%	18	38%
	Hospital day-cases, total and selected diagnoses	3	14	na	60	78%	11	23%
Quality of Care	Prevalence and incidence rate of hospital-acquired infections (% of patients hospitalised)	1	4	3	50	90%	21	47%
	Vaccination coverage in children	2	1	na	52	85%	29	66%
	Rate of patients with colorectal tumour receiving chemotherapy whose treatment started within two months following surgery	2	35	na	65	68%	8	18%
	Screening rates for selected cancers (breast, cervical, colon)	4	5	na	51	84%	29	66%
Equity	Percentage of households experiencing high levels/catastrophic of out-of-pocket health expenditures	1	7	3	68	63%	14	33%
	Self-reported unmet need for medical care (total by reason: cost, waiting time, distance)	2	2	na	68	62%	20	48%
	Private household out-of-pocket expenditure as a proportion of total health expenditure	3	19	na	67	61%	10	24%
Health Status	Life expectancy	1	2	1	52	90%	35	74%
	Healthy Life Years (HLY)	2	3	1	51	82%	26	62%
	Infant mortality rate	3	1	4	50	78%	30	77%
Health Determinants	At risk of poverty or social exclusion rate	1	34	na	45	89%	7	18%
	Body Mass Index	2	3	2	44	82%	17	47%
	Prevalence of different smoking status, self-reported	2	4	1	43	84%	16	44%
	Unemployment rate	4	56	na	45	78%	5	14%

*na* not available

^a^the statistical rank of the respective indicator based on the HSPA domain or headline level per HSPA domain frequency. If there are duplicate values in the ranking, these are given the same rank

^b^$$ HSPA\ {domain\ frequency}_{i\ d}=\sum \limits_{r=1}^N{x}_r $$,

^c^$Headline\ level\  per\ {HSPA\ frequency}_{i\ d\ h}=\sum \limits_{r=1}^N{y}_r$where **i** is indicator ID, **d** is domain name (access / efficiency / equity / quality of care / health status / health determinants), **h** is headline level, **x** is received score for domain, **y** is received score for headline level, **r** is questionnaire ID

**Table 5:** Top three indicators ranked by headline frequency

Domain	Name of indicator	Ranking by^a^			Frequencies				Availabilities		
		Headline	HSPA domain	Individual preferences	HSPA domain^b^		Headline^c^		ECHI / Eurostat	OECD	WHO-EUR
					N responded	assessed for domain %	N responded	assessed for headline per HSPA domain %			
Access	Share of population covered by health insurance	1	1	1	69	96%	43	65%	✓	✓	–
	Accessibility to acute care	2	3	3	66	92%	26	43%	–	–	–
	Self-reported unmet need for medical care (total by reason: cost, waiting time, distance)	3	2	na	68	91%	25	40%	✓	✓	–
Efficiency	Total health care expenditure by all financing agents (total, public and private sectors)	1	6	1	54	76%	20	49%	✓	✓	✓
	Hospital beds	2	2	na	61	79%	18	38%	✓	✓	✓
	Vaccination coverage in children	2	23	na	52	54%	18	64%	✓	✓	✓
	Current health care expenditure (CHE) by all financing agents (total, public and private sectors)	4	8	na	54	67%	17	47%	✓	✓	✓
Quality of Care	Vaccination coverage in children	1	2	na	52	85%	29	66%	✓	✓	✓
	Infant mortality rate	2	13	na	50	72%	26	72%	✓	✓	✓
	Maternal mortality rate	3	5	na	48	88%	25	60%	–	✓	✓
Equity	Share of population covered by health insurance	1	4	na	69	57%	27	69%	✓	✓	–
	Self-reported unmet need for medical care (total by reason: cost, waiting time, distance)	2	2	na	68	62%	20	48%	✓	✓	–
	Accessibility to acute care	3	8	na	66	52%	19	56%	–	–	–
Health Status	Life expectancy	1	1	1	52	90%	35	74%	✓	✓	✓
	Infant mortality rate	2	3	4	50	78%	30	77%	✓	✓	✓
	Healthy Life Years (HLY)	3	2	1	51	82%	26	62%	✓	✓	✓
Health Determinants	Share of population covered by health insurance	1	7	na	69	46%	18	56%	✓	✓	✓
	Life expectancy	2	17	na	52	50%	18	69%	✓	✓	✓
	Body Mass Index	3	2	2	44	82%	17	47%	✓	✓	✓

*na* not available

^a^ the statistical rank of the respective indicator based on the HSPA domain or headline level per HSPA domain frequency. If there are duplicate values in the ranking, these are given the same rank. (✓) indicator available, (−) not available

^b^$$ HSPA\ {domain\ frequency}_{i\ d}=\sum \limits_{r=1}^N{x}_r $$,

^c^$Headline\ level\  per\ {HSPA\ frequency}_{i\ d\ h}=\sum \limits_{r=1}^N{y}_r$where **i** is indicator ID, **d** is domain name (access / efficiency / equity / quality of care / health status / health determinants), **h** is headline level, **x** is received score for domain, **y** is received score for headline level, **r** is questionnaire ID

**Table 6:** Top three listed headline indicators ranked by individual preferences

Domain	Name of indicator	Ranking by^b^			Frequencies		Availabilities		
		Individual preferences	HSPA domain	Headline	N responded	assessed by individual preferences as headline %	ECHI / Eurostat	OECD	WHO-EUR
Access	Share of population covered by health insurance^a^	1	1	1	25	36%	✓	✓	–
	Reported waiting times for access o specialist (care)	2	5	8	24	17%	–	–	–
	Accessibility to acute care	3	3	2	22	18%	–	–	–
	Waiting times for elective surgeries	3	8	31	22	18%	–	✓	–
Efficiency	Average length of stay (ALOS), total and selected diagnoses	1	1	39	22	14%	✓	✓	✓
	Total health care expenditure by all financing agents (total, public and private sectors)^a^	1	6	1	22	14%	✓	✓	✓
	Health expenditure per capita in PPP (purchasing power parities) in relation to life expectancy at birth	2	21	34	20	20%	~	~	~
	Number of surgical operations and procedures	4	10	55	16	25%	✓	✓	✓
Quality of Care	Hospital Standardized Mortality Ratio (HSMR)	1	11	12	24	25%	➔	➔	–
	Ambulatory Care Sensitive Conditions (ACSC) Hospitalization Rate	2	8	15	22	14%	–	✓	–
	Prevalence and incidence rate of hospital-acquired infections (% of patients hospitalised)^c^	3	1	4	20	25%	–	–	–
Equity	GINI coefficient (income distribution)	1	20	48	17	35%	✓	✓	✓
	Geographic distribution of doctors: Physicians density in predominantly urban and rural regions	2	8	7	17	18%	~	~	~
	Percentage of households experiencing high levels/catastrophic of out-of-pocket health expenditures^a^	3	1	7	17	12%	–	✓	–
	Self-reported/perceived general health	3	20	15	17	12%	✓	–	–
Health Status	Healthy Life Years (HLY)	1	2	3	22	18%	✓	✓	✓
	Life expectancy^a^	1	1	2	22	18%	✓	✓	✓
	Avoidable mortality rate: amenable and preventable deaths	2	8	5	20	15%	✓	–	–
	Infant mortality rate	4	3	1	18	28%	✓	✓	✓
Health Determinants	Prevalence of different smoking status, self-reported^a^	1	2	4	22	36%	✓	✓	✓
	Body Mass Index^a^	2	2	3	19	21%	✓	✓	✓
	Opportunities for education: Participation in early childhood education	3	7	24	17	18%	✓	✓	–
	Overall experience of life: Life satisfaction	3	20	8	17	18%	✓	✓	–

^a^robust indicator with the lowest sum of all three rankings

^b^the statistical rank of the respective indicator based on the HSPA domain or headline level per HSPA domain frequency. If there are duplicate values in the ranking, these are given the same rank. (✓) indicator available, (~) not ready-made and needs to be calculated, (−) not available, (➔) indicator available just for selected diagnoses

^c^provided by ECDC

#### Access

Of 66 experts 43 (65%) assessed share of population covered by health insurance as the top-level headline indicator in this domain. The importance of this indicator was also highlighted by rankings given through individual preferences from 9 out of 25 experts (36%). In Table 5, accessibility to acute care ranks second, assessed by 26 out of 61 (43%) respondents, and 25 out of 62 (40%) assessed self-reported unmet need which thus ranks third. Both indicators are also listed in Table 4, but in reverse order. Interestingly, process indicators on waiting times for access to specialist care and for elective surgeries only received high priority on second and third rank when listed individually by respondents.

#### Efficiency

The ranking shows that mostly input indicators were ranked high while pushing full efficiency measures (input/output/outcome metric) down. Top three headline indicators in Table 5 are largely measures of costs such as 1) total healthcare expenditure by all financing agents which is identified as a “top-level” indicator, 20 out 41 (49%), and 3) current healthcare expenditure by all financing agents (total, public and private sectors), 17 out 36 (47%). Among the top two are hospital beds, 18 out of 48 (38%) and vaccination coverage in children, 18 out of 28 (64%) due to a tie in ranking. In ranking top three preferred headline indicators per HSPA domain individually at the end of the survey (Table 6), only four respondents out of 20 (20%) reported an input to outcome measure as top two, e.g. health expenditures per capita in PPP (purchasing power parities) in relation to life expectancy as preferred top one.

#### Quality of care

1) vaccination coverage in children, 29 out of 44 (66%), 2) infant mortality rates, 26 out of 36 (72%), and 3) maternal mortality rate, 25 out of 42 (60%) were rated as top headline indicators in Table 5. On the contrary, results from the individual ranking preferences show 1) hospital standardized mortality ratio, 6 out of 24 (25%), 2) ambulatory care sensitive conditions hospitalization rate, 3 out of 22 (14%), and 3) prevalence and incidence rate of hospital-acquired infections (HAI), 5 out of 20 (25%) were named as the most top three headline indicators, see Table 6. Besides, two process indicators rate of patients with colorectal tumour receiving chemotherapy and screening rates for selected cancers were allocated on second and third rank for the quality domain in Table 4. However, a closer look at the different rankings reveals that the HAI rate, a process indicator, is in the upper bound of all rankings and consequently identified as “top-level” headline indicator for this domain.

#### Equity

The top three headline indicators from Table 5 are 1) share of population covered by health insurance, 27 out of 39 (69%), 2) self-reported unmet need for medical care, 20 out of 42 (48%), and 3) accessibility to acute care, 19 out of 34 experts (56%). These indicators were also scored headline in the domain of access indicating that experts pertained to the concept of equity in access rather than equity in outcomes. Nevertheless, when the rankings by HSPA domain (Table 4) and individual preferences (Table 6) are considered percentage of households experiencing high levels/catastrophic of out-of-pocket health expenditures, 43 out of 68 experts (63%) results as the “top-level” headline indicator.

#### Health status

1) life expectancy, 35 out of 47 (74%), 2) infant mortality rate, 30 out of 39 (77%), and 3) healthy life years, 26 out of 42 (61%) received the highest scores in Tables 4 and 5 (different order). These results are also mirrored by results of the individual ranking in Table 6 which are only complemented by avoidable mortality rate deemed important for the second rank and life expectancy being the “top-level” headline indicator.

#### Health determinants

Headline results in Table 5 overlapped with headline access, equity and health status indicators: 1) share of population covered by health insurance, 18 out of 32 (56%), 2) life expectancy, 18 out of 26 (69%), and 3) body mass index, 17 out of 36 (47%). Clearly and more accurately, HSPA domain and individual rankings reveal the importance of lifestyle specific aspects which rank among the top 3: prevalence of different smoking status, 8 out of 22 (36%), and participation in early childhood education, 3 out of 17 (18%) were attributed a greater importance, see Tables 5 and 6. Based on these smoking status and BMI result as being “top-level” headline indicators for health determinants.

Table 5 also shows the availability of the 19 selected headline indicators in the most common health data repositories as per September 2017. 84% (16/19) of top three headline indicators are available in the ECHI/Eurostat database, 84% (16/19) in the OECD database, and 63% (12/19) in the WHO-EUR data gateway.

### Headline indicator criteria

Respondents’ average ratings reflecting the importance of the 11 criteria for a headline indicator are summarised in Table 7. Forty-nine percent of those surveyed (*n* = 36) responded to this question. Overall, 9 out of 11 criteria were rated as important (1) or probably important (2), the top three criteria being validity, reliability and that an indicator needs to be clear and easy to communicate & interpret. While validity was ranked as most important (Mean = 1.32, SD = 0.48) by participants affiliated with a governmental institution, for researchers the criterion clear and easy to communicate & interpret had the highest importance (Mean = 1.40, SD = 0.66). Indicators which are routinely available was most important for healthcare providers completing the survey (Mean = 1.00, SD = 0.00). Regarding international comparability, results show a statistically significant difference (χ2 test, *p* < 0.001) between respondents affiliated with governmental institutions (rank = 3, Mean = 1.47, SD = 0.77) and researchers (rank = 6, Mean = 1.69, SD = 1.03).

**Table 7:** Priority ranking of headline indicator criteria by respondents’ affiliation

	Total (*N* = 36)				Governmental or other public institution (*N* = 19)			Health care provider (*N* = 2)			Research institution (*N* = 15)		
Criterion	Mean	SD	Rank	*P*-value *	Mean	SD	Rank	Mean	SD	Rank	Mean	SD	Rank
Validity	1.36	0.54	1	0.769	1.32	0.48	1	1.50	0.71	2	1.40	0.75	2
Reliability	1.44	0.61	2	0.986	1.44	0.62	2	1.50	0.71	3	1.44	0.51	3
Clear and easy to communicate & interpret	1.50	0.56	3	0.466	1.53	0.61	4	2.00	0.00	7	1.40	0.66	1
International comparability	1.65	0.79	4	0.000	1.47	0.77	3	3.00	1.41	9	1.69	1.03	6
Actionability	1.70	0.62	5	0.936	1.68	0.58	6	2.50	0.71	8	1.63	0.60	5
Policy relevance	1.72	0.70	6	0.231	1.67	0.69	5	1.50	0.71	4	1.81	0.82	7
Routine availability	1.72	0.61	7	0.830	1.68	0.58	7	1.00	0.00	1	1.81	1.45	8
Coherent and balanced overall	1.72	0.66	8	0.654	1.84	0.69	9	1.50	0.71	5	1.60	0.62	4
International feasibility	1.92	1.04	9	0.011	1.68	1.11	8	3.50	0.71	10	2.00	0.63	9
Farreaching	2.31	0.86	10	0.325	2.28	0.67	10	1.50	0.71	6	2.44	0.63	10
Minimum number of indicators	2.64	1.27	11	0.805	2.53	1.17	11	3.50	0.71	11	2.67	0.63	11

*SD* standard deviation

* chi-square test

## Discussion

This study identified important and relevant “headline” indicators for HSPA that have potential to focus and improve cross-country comparisons. Experts’ perceptions were also obtained about the most relevant criteria that should underline the prioritisation of indicators. The main strength of our study lies in the systematic and comprehensive approach adapted in mapping the current EU-relevant HSPA indicator landscape. To enhance cross-country knowledge exchange, this was complemented by other international initiatives. Currently, no similar consolidated indicator inventory does exist. To strengthen further research in the area, the relevant database containing the full raw and consolidated list of indicators has now been made available at websites of HS&I and the Medical University of Vienna [79].

Our results highlight several main points for further considerations, especially in the light of some of the comments respondents provided.

Firstly, the distribution of available indicators is unbalanced and dominated by areas such as quality of care, health status and determinants of health which largely overlap. These have been driven by policy and research with the aim to improve health information quality and availability in the area of public health (e.g. DG Santé-ECHI, OECD HCQI). This is also reflected in the recent call for further progress on the development and use of patient reported outcome measures (PROMs) which besides of self-reported health as equity indicator, did not receive top priority for headline possibly due to lack of data availability [27, 40, 80–83]. Overall, results from Table 6 indicate that the distribution of types of indicators (e.g. outcome, process and structural measures) is rather balanced across HSPA domains, but not within. Furthermore, some indicators refer to macro level areas (e.g. health expenditure) whereas others relate to more meso level aspects (e.g. hospital sector). Again, these might be explained by the data availability as a driver for respondent’s assessment. As for the indicator vaccination coverage in children, respondents underlined its high relevance (Q11) as well as suggestions to break down by socioeconomic status (Q28). Regarding the maternal mortality rate it was mentioned that due to incomplete data this indicator is less suitable for evaluation purposes (Q11) and remains debatable whether it qualifies as a good headline indicator. Lifestyle indicators (e.g. obesity, smoking and alcohol consumption) received high priority on headline level only through individual preferences and when assessed for specific HSPA domain as many respondents were experts in epidemiology and health determinants. Overall the top indicator list benefited from looking at all three types of frequency rankings. For example, smoking status would not have made it to the list although it is a key health determinant while life expectancy would have been inaccurately mapped as a health determinant.

Secondly, efficiency indicators which combine outcome with input measures are rare, they are not often used and appear not well understood. While both, the EU Health Strategy “Together for Health” [84] and the official EC communication [4], referred to the high importance of efficiency, there is much work still to be done in developing metrics that are able to compare health system efficiency across countries [16, 85]. This reflects difficulties in agreeing on information standards and protocols and defining adequate outcome metrics to be combined with input metrics. Our findings suggest that more multidisciplinary work is needed to enhance efforts in making accurate, cross-country comparable efficiency indicators available for comprehensive HSPA [86]. This is echoed in the 2018 work programme of the Expert Group on HSPA, a forum where MSs exchange experiences on the use of HSPA at national level and which looks specifically at tools and methodologies to assess efficiency [87].

Thirdly, our findings are in line with the global priority areas reflected in the Sustainable Development Goals from 2015 [88]. Considering access to healthcare, it confirms the ultimate importance of financial protection in achieving comprehensive universal health insurance coverage. When looking at comments of survey participants, several related aspects were addressed. For example, one respondent said that due to mandatory full coverage of the population in some countries this indicator might not be a suitable measure of performance (Q28). Also some concerns on self-reported unmet need were raised mirroring a widespread scepticism towards self-assessed health [14]. Due to its huge differences between the results from the European Union Statistics on Income and Living Conditions (EU-SILC) and the Health Interview Survey (HIS) respondents indicate that this subjective indicator is difficult to interpret (Q15, Q60), and thus misses to provide actionable information which weakens suitability for international comparison (Q3). Others indicated, that the headline suitability for the indicator accessibility to acute care also depends first on a clear definition and further on countries’ health system design where a decline or failure is unmeasurable because it is incorporated into law, e.g. percentage of people who can reach primary, emergency and maternity care services is guaranteed within 20 min (Q26). It was suggested to look at the distribution across geographical areas, in relation to deprivation index to increase actionability of this indicator (Q36). Further, one respondents said that “substantial amount of analysis and decisions regarding health are taken at sub-national level and many policies and investments that affect population health are set regionally” (Q30). This reflects the importance the availability of high quality regional level indicators suitable for performance assessment on individual country level. Finally, we were able to show the feasibility of indicator priority elicitation across many stakeholders and the potential to make priority setting more evidence-based, as required in a recent analysis of priority setting methods in health information [89]. With this survey we were able to identify potential so-called “top-level” headline indicators that appear in all, HSPA domain, headline level and individual preference frequency which not only matter to policy makers, but also to people. We believe that the level of coverage of risks is important to people, mirrored also by the indicator of private / out of pocket payments listed in Table 6. Even though many criteria need to be considered and criteria priority vary depending on the targeted audience, headline information on health systems is crucial. Nevertheless, the applied method may also be used at country level and even at provider level as many MSs have a regionalized health system. A prioritized set of agreed and robust indicators might serve decision makers information needs to compare and potentially benchmark regional health systems which can encourage the provision of good quality data from stakeholders [90].

### Limitations

This study has a number of limitations.

Firstly, the overall response rate was moderate. This may be explained by the survey length and the unprecedented approach to define headline indicators in the health sector. In the 2nd stage, a representative response rate from 22 EU MSs was achieved. While no response was received from Denmark, Estonia, Greece, Liechtenstein, Malta and Poland in the 2nd stage, representatives from Denmark and Malta participated in the 1st stage of the euHS_I survey. Due to the small sample size of healthcare providers, however, caution must be applied in regard to the representativeness of our findings in terms of all different stakeholder groups.

Secondly, validation of results, i.e. assessment based on the ranked indicator criteria such as data availability across MSs, and the investigation of collinearity between the shortlisted indicators has not been performed at this stage. At the same time, 47 out of 95 (49.5%) included indicators in the 2nd stage and 84% identified as headline are coming from ECHI which have predefined standards and are mostly fed by Eurostat data. Further validation of the results regarding their usefulness in assessing system performance for policy makers through qualitative interviews is also in progress.

Thirdly, our sample size does not allow for further statistical analyses exploring potential differences in responses across participants from different countries and across groups with different types of experience and potential adjustment to our rankings according to these. A more comprehensive coverage of experts and multiple responses from individual countries, however, would have required substantially larger research resources that were not available for the current project.

Fourthly, there are likely several biases. Expert background from authors is health economics, health services research/health policy, and mental health which might have introduced a bias towards the outcome of the process in consolidating the indicators inventory for overlaps. This could have caused some unintended ambiguous indicator groupings by theme chapters. Therefore, the provided explanatory information to survey participants and the published full inventory [79] is very essential in increasing the transparency of this research. Furthermore, the expertise of respondents has apparently influenced the outcomes of the study (e.g. a high number of health economics in the 1st stage and a low number of experts on long-term care or pharmaceuticals in both stages). The basket of identified headline indicators does not contain any indicator on long term care although it is a significant contributor to health system expenditures. It appears that long-term care is not often not seen as part of a health system because it belongs to the broader social policy agenda in many countries. Surprisingly, indicators of pharmaceutical care also didn’t make it to the top list although the cost pressure coming from these products is high. At the same time indicators in these areas are given operational or explanatory function. This indicates the awareness of respondents that they are important for a more in-depth analysis of specific policy aspects. Our results will be validated with policy-makers in a qualitative approach to reflect all topical health policy aspects which aims to broaden our understanding of the relevance of indicators and their importance. Finally, while participants had good pre-knowledge and thorough expertise with HSPA indicators it is likely that subjective bias may have influenced individual responses.

### Recommendations for future research

In line with Europe 2020’s headline indicators [17], we suggest the establishment of a similar structure in the area of HSPA. For example, an electronic repository could be created featuring headline and lower level indicators as classified to provide timely benchmarks following the example of the macro-economic database AMECO of EC’s Directorate General for Economic and Financial Affairs [91]. Maintained and up-dated timely with short-term forecasts of key indicators, such an inventory would be indispensable for analysis and reporting. In fact, it would improve the overall value of information provided [14].

Our research has raised many questions in need of further investigation. Further research should be done to investigate where improved information through new indicator development would lead to biggest improvements in decision-making, measured for example by burden of disease. Likewise, there is abundant room for further progress in determining the suitability and sufficiency of proxy indicators for certain purposes. More broadly, this would require addressing the issue of costs of collecting indicators and assessing their “value of information” to determine the incremental benefits [14].

## Conclusions

The results of our research may provide a blueprint for most important and relevant “headline” indicators which may be used in framing and describing the performance of a health system in the EU context at a first glance. This should eventually lead to an informative refinement of the ECHI shortlist. Our study has proposed structured priority elicitation across many stakeholders and contributes to evidence-based, multi-sectorial priority setting methods. Moreover, our findings encourage more multidisciplinary work to increase the availability of accurate indicators for cross-country comparisons in the area of efficiency to promote comprehensive HSPA.

## Additional files

Additional file 1: List of excluded rather country-specific indicators. (XLSX 26 kb)

Additional file 2: Framework and definitions of HSPA domains based on EuroREACH [10]. (DOCX 88 kb)

Additional file 3: Explanatory information of presented headline indicators per HSPA domain. (DOCX 41 kb)

## Abbreviations

ECHI: European Core Health Indicators
EU: European Union
EuroREACH: A handbook to access health care data for cross country comparisons of efficiency and quality
EXPH: Expert panel on effective ways of investing in health
HCQI: Health care quality indicators
HSP: Health system performance
HSPA: Health system performance assessment
MS: Member State
OECD: Organisation for economic co-operation and development
Q: questionnaire
SDI: Sustainable development indicators
WHO-EUR: World Health Organisation Regional Office for Europe
