# Supplementary material for: Headline indicators for monitoring the performance of health systems: findings from the european Health Systems_Indicator (euHS_I) survey
Source: Arch Public Health. 2018 Jun 28;76:32. doi: 10.1186/s13690-018-0278-0 (PMC6022696; doi:10.1186/s13690-018-0278-0)
Supplement: Supplementary file 2 — Framework and definitions of HSPA domains based on EuroREACH [10]. (DOCX 88 kb) [file 13690_2018_278_MOESM2_ESM.docx]

## Additional file 2: Framework and definitions of HSPA domains based on EuroREACH [1]


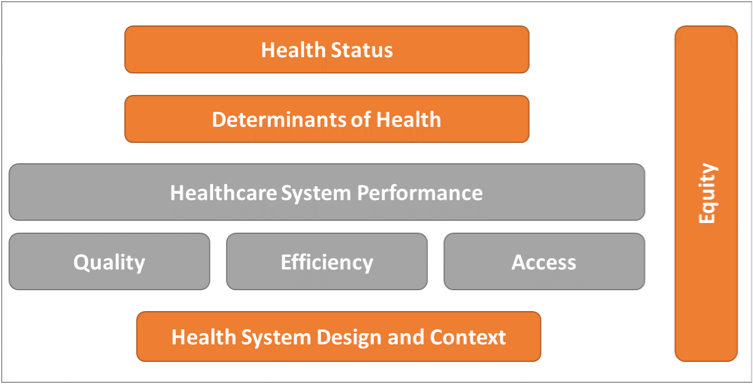


| Domain | Definition |
| --- | --- |
| Health Status | The World Health Organization (1948) defines health as “a state of complete physical, mental, and social well-being and not merely the absence of disease or infirmity”. Thus health encompasses a broad spectrum of dimensions and is impacted by a multitude of different factors, some inherent and other external to the individual. Different health status measures are used in the literature, from subjective measures (self-assessed health) to more objective measures of health (biological markers). |
| Health Determinants | Determinants refer to non-health care factors that may influence health. These cover socio-economic factors, environmental conditions, lifestyle and/or life course events which may shape or disrupt health (Arah et al., 2006). |
| Healthcare System Performance | Performance is interpreted differently by different stakeholders. A common definition is “the degree of achieving objectives set”. According to Baldrige, performance refers to results obtained from processes, products, and services. Performance measurement is crucial for improving health systems functioning. Without measurement, it is difficult to identify good and bad delivery practice or high/low quality practitioners, to tailor health system reforms, to protect patients or payers, or to make the case for improving quality, efficiency by investing in health care. Measurement is central to accountability to citizens, patients and payers for health system actions and outcomes.  In the euHS_I survey, “Health System Performance” would be broken down into 5 columns:   - Access(inability) - Quality: Effectiveness - Quality: Patient centredness - Quality: Safety - Efficiency |
| Quality | Quality in the euHS_I survey refers primarily to “The degree to which health services for individuals and populations increase the likelihood of desired health outcomes and are consistent with current professional knowledge”, i.e. the “effectiveness” component of quality. While there is an overall consensus that quality encompasses several components, there is no agreement on these, e.g. some would include “accessibility” which OECD and we list separately. Following OECD, we list “patient-centeredness” and “safety” as relevant other components. |
| Efficiency | Efficiency in the euHS_I survey takes a comparative system perspective and is defined in three dimensions. **Technical efficiency** refers to the relationship between outputs, e.g. bed-days, discharges and inputs, e.g. nurses and beds, cost). **Allocative efficiency** refers to the allocation of funds between care sectors, e.g. prevention, primary care, long-term care to ensure maximum outcomes levels. **Cost effectiveness** refers to the ratio of valued outputs to inputs. Although there have been several attempts to clarify the nature of inputs and outputs, there is no consistent approach. In particular, there is considerable variation as to what the valued outputs are, including: volume of care, quality of care, levels of quality, ‘performance’ and health improvement, reflecting the lack of clarity as to the concept of ‘valued outputs’. |
| Access | Access in the euHS_I survey refers to the WHO (2000) definition indicating “Ensuring access to care based on need and not ability to pay”. Definitions for access are very different in type and form. While some are very specific and precise, others are broad and vague. Most of the definitions do not clearly define concepts such as 'ease' or what entails 'possibility' of accessing health care. The differences show the lack of agreement and challenge in defining a concept such as access. |
| Equity | Equity (in outcomes) in the euHS_I survey refers to “The extent to which disparities or inequalities in health among subgroups of the population are minimized”. Equity is not defined by preferences but by values or judgments. Although several definitions leave an open interpretation to equity allowing for several perspectives, the majority of European countries take an egalitarian viewpoint of social justice, e.g. that “health (care) should be distributed according to need” and “financed according to the ability of pay”. “Access” and “need” are terms which are difficult to conceptualize and measure. |
| Health System Design and Context | Understanding institutional characteristics of health care systems is essential for carrying out comparative analysis of policies and strategies to improve system performance. System features such as regulation of health care supply and demand, incentives for providers, level of public/private funding or the extent of coverage, among others, have a direct impact on system functioning and hence on its performance. Researchers and policymakers need to understand the strengths and limitations of different systems including their own by analyzing countries according to common institutional characteristics or policies. There are several different ways a "health system" or "health care" can be defined and conceptualized (see Frameworks) which will ultimately determine the focus of organizational and institutional analysis. |

1. Hofmarcher M, Smith P. The Health Data Navigator. Your toolkit for comparative performance analysis. A EuroREACH product. 2013. <http://www.healthdatanavigator.eu/HDN_Toolkit_Final.pdf>. Accessed Oct 15 2015.
