# Supplementary material for: Headline indicators for monitoring the performance of health systems: findings from the european Health Systems_Indicator (euHS_I) survey
Source: Arch Public Health. 2018 Jun 28;76:32. doi: 10.1186/s13690-018-0278-0 (PMC6022696; doi:10.1186/s13690-018-0278-0)
Supplement: Supplementary file 3 — Explanatory information of presented headline indicators per HSPA domain. (DOCX 41 kb) [file 13690_2018_278_MOESM3_ESM.docx]

# Additional file 3: Explanatory information of presented headline indicators per HSPA domain

| **Domain** | **Name of indicator** | **Explanatory info for survey** |
| --- | --- | --- |
| **Access** | Share of population covered by health insurance * | Percentage of population covered by i) Government/ social health insurance, ii) private health insurance. |
|  | Accessibility to acute care * | Percentage of people who can REACH primary, emergency and materinity care services within 15/30 minutes |
|  | Self-reported unmet need for medical care (total by reason: cost, waiting time, distance) * | Disaggregated by sex, by age group (total, 18-64, 65+), by education level (ISCED 0-1, 2, 3-4, 5-6) and by income quintile. Proportion of persons with self-declared unmet needs for medical care services due to either financial barriers, waiting times or traveling distances. |
|  | Reported waiting times for access to specialist (care) | No attempt was made to provide a single definition; instead following definitions are considered as examples:  Waiting time of more than two weeks to get an appointment with a specialist (% of population asking an appointment).  Waited 2 months or longer for specialist appointment (base: needed to see specialist in past 2 years) (by two income categories). Waiting time longer than 1 month for first contact in ambulatory mental health centre (% of pop with contact in ambulatory mental health centre). Waiting time, referral to treatment: Doctors report patients often experience long wait times to receive treatment after diagnosis (e.g. cancer treatment delays). Percentage of patients treated within 18 weeks. |
|  | Waiting times for elective surgeries | Average inpatient waiting time for elective (i.e. non-urgent) surgeries of Percutanerous Transluminal Coronary Angioplasty (PTCA), hip replacement and cataract operation, measured in number of days. Elective surgery is defined as when surgery is necessary, but the timing of the procedure can be scheduled and the patient can be sent home. |
| **Efficiency** | Total health care expenditure by all financing agents (total, public and private sectors) | Total health expenditure for total, public, and private sectors expressed in following units: i) in Purchasing Power Standard (PPS) per capita, ii) as percentage of gross domestic product (GDP), iii) in millions of Purchasing Power Standard (PPS).  ‘Total expenditure on health measures the final consumption of health goods and services (i.e. current health expenditure, CHE) plus capital investment in health care infrastructure. This includes spending by both public and private sources on medical services and goods, public health and prevention programmes and administration. To compare spending levels between countries, per capita health expenditures are converted to a common currency (US dollar) and adjusted to take account of the different purchasing power of the national currencies, in order to compare spending levels’. |
|  | Hospital beds * | The total number of hospital beds per 100,000 inhabitants. (total number, acute care, psychiatric care, long-term care) |
|  | Vaccination coverage in children | Disaggregated by age.  Percentage of infants who have been fully vaccinated against important infectious childhood diseases according to national vaccination schemes. |
|  | Current health care expenditure (CHE) by all financing agents (total, public and private sectors) | Current health expenditure (CHE) for total, public, and private sectors, expressed in following units: i) in Purchasing Power Standard (PPS) per capita, ii) as percentage of gross domestic product (GDP), iii) in millions of Purchasing Power Standard (PPS).  Current health expenditure measures the final consumption of health goods and services without capital investment in health care infrastructure. This includes spending by both public and private sources on medical services and goods, public health and prevention programmes and administration. To compare spending levels between countries, per capita health expenditures are converted to a common currency (US dollar) and adjusted to take account of the different purchasing power of the national currencies, in order to compare spending levels’. |
|  | Average length of stay (ALOS), total and selected diagnoses | Total population age-standardized, for selected diagnoses; by type of care (acute, mental and rehabilitation hospitals) disaggregated by age group (0-64, 65+), by sex. In-patient average length of stay (in days) , for selected diagnose (ISHMT code 0000 = ICD-10 codes A00-Z99 excluding V,W,X & Y codes and healthy newborns Z38, e.g. cancers, AMI, normal delivery, ).  Average length of stay (ALOS) is computed by dividing the total number of in-patient hospital days , in all hospitals, counted from the date of admission to the date of discharge by the total number of discharges (including deaths) in all hospitals during a given year. A hospital day (or bed-day or in-patient day) is a day, during which a person admitted as an in-patient, is confined to a bed and stays overnight in a hospital. Day-cases (patients formally admitted for a medical procedure or surgery in the morning and discharged before the evening) are excluded. Patients admitted with the intention of discharge on the same day, but who subsequently stay in hospital overnight, are included. |
|  | Health expenditure per capita in PPP (purchasing power parities) in relation to life expectancy at birth | Total health care expenditure by all financing agents (total, public and private sectors) per capita in PPP defined as the final consumption of health goods and services (i.e. current health expenditure, CHE) plus capital investment in health care infrastructure. This includes spending by both public and private sources on medical services and goods, public health and prevention programmes and administration  IN RELATION to  Life expectancy at birth defined the average number of years of life remaining if a group of persons at that age were to experience the mortality rates for a particular year over the course of their remaining life. |
|  | Number of surgical operations and procedures | The number of surgical operations and procedures performed in hospitals, inpatient surgery, age-standardized per 100,000 population, by sex,  For following categories: PTCA (Percutaneous transluminal coronary angioplasty); Hip Replacement, Cataract, Tonsillectomy, Coronary Artery Bypass Graft, Laparoscopic Cholecystectomy, Repair of Inguinal Hernia, Caesarean Section, Total Knee Replacement, Partial Excision of Mammary Gland, Total Mastectomy, non-conservative breast surgery, Hysterectomy in uterine cancer, Hysterectomy without uterus cancer diagnosis, Adenoidectomy and/or tonsillectomy, Prostatectomy with prostate cancer/benign prostatic hyperplasia, Kidney transplants |
| **Quality of Care** | Vaccination coverage in children * | Disaggregated by age.  Percentage of infants who have been fully vaccinated against important infectious childhood diseases according to national vaccination schemes. |
|  | Infant mortality rate | The ratio of number of deaths of children under one year of age to the number of live births. The value is expressed per 1000 live births. |
|  | Maternal mortality rate | The maternal mortality ratio is the annual number of female deaths from any cause related to or aggravated by pregnancy or its management (excluding accidental or incidental causes), during pregnancy and childbirth or within 42 days of termination of pregnancy, irrespective of the duration and site of the pregnancy, per 100 000 live births, for a specified year. |
|  | Hospital Standardized Mortality Ratio (HSMR) | This indicator examines the ratio of the actual number of in-hospital deaths in a region or hospital to the number that would have been expected based on the types of patients a region or hospital treats. The HSMR is calculated by dividing the actual number of in-hospital deaths by the expected number of in-hospital deaths, for conditions accounting for about 80% of inpatient mortality. |
|  | Ambulatory Care Sensitive Conditions (ACSC) Hospitalization Rate | Disaggregated by sex, age, condition.  Avoidable hospitalization rate / Emergency admissions to hospital (indirectly standardised rate per 100,000 population) of persons with an ambulator care sensitive condition (ACSC).  ACSCs are conditions for which effective management and treatment should prevent admission to hospital. They can be classified as: chronic conditions (e.g. asthma, COPD, Congestive heart failure, diabetes, etc.), where effective care can prevent flare-ups; acute conditions (ear/nose/throat infections, kidney/urinary tract infections, heart failure, among others), where early intervention can prevent more serious progression; and preventable conditions, where immunisation and other interventions can prevent illness (Ham et al 2010).  Source: http://www.kingsfund.org.uk/sites/files/kf/field/field_publication_file/data-briefing-emergency-hospital-admissions-for-ambulatory-care-sensitive-conditions-apr-2012.pdf |
|  | Prevalence and incidence rate of hospital-acquired infections (% of patients hospitalised) * | Incidence of hospital-acquired MRSA infections (/1000 hospital stays).  Incidence of healthcare associated infection (HSAI) – C. difficile.  An infection is considered an HAI if all elements of a CDC/NHSN (Centers for Disease Control and Prevention/National Healthcare Safety Networks) site-specific infection criterion were first present together on or after the 3rd hospital day (day of hospital admission is day 1). For an HAI, an element of the infection criterion may be present during the first 2 hospital days as long as it is also present on or after day 3. All elements used to meet the infection criterion must occur within a timeframe that does not exceed a gap of 1 calendar day between elements’ |
| **Equity** | Share of population covered by health insurance | Percentage of population covered by i) Government/ social health insurance, ii) private health insurance. |
|  | Self-reported unmet need for medical care (total by reason: cost, waiting time, distance) * | Disaggregated by sex, by age group (total, 18-64, 65+), by education level (ISCED 0-1, 2, 3-4, 5-6) and by income quintile. Proportion of persons with self-declared unmet needs for medical care services due to either financial barriers, waiting times or traveling distances. |
|  | Accessibility to acute care | Percentage of people who can REACH primary, emergency and materinity care services within 15/30 minutes |
|  | GINI coefficient (income distribution) | Gini index measures the extent to which the distribution of income (or, in some cases, consumption expenditure) among individuals or households within an economy deviates from a perfectly equal distribution. A Lorenz curve plots the cumulative percentages of total income received against the cumulative number of recipients, starting with the poorest individual or household. The Gini index measures the area between the Lorenz curve and a hypothetical line of absolute equality, expressed as a percentage of the maximum area under the line. Thus a Gini index of 0 represents perfect equality, while an index of 100 implies perfect inequality. |
|  | Geographic distribution of doctors: Physicians density in predominantly urban and rural regions | Density per 1 000 population |
|  | Percentage of households experiencing high levels/catastrophic of out-of-pocket health expenditures * | nA (Percentage of households experiencing high levels of/ catastrophic out-of-pocket health expenditures using different thresholds by age and income quintiles). |
|  | Self-reported/perceived general health | Disaggregated by by sex, age-group (15-64, 65+), educational level (ISCED 0-1, 2, 3-4, 5-6), by income quintile (gap q1/q5).  Proportion of persons who assess their health to be very good or good. |
| **Health Status** | Infant mortality rate * | The ratio of number of deaths of children under one year of age to the number of live births. The value is expressed per 1000 live births. |
|  | Life expectancy * | Total population, disaggregated by sex and by educational attainment.  Life expectancy at birth, ages 1, 15, 45 and 65 years represents the average number of years of life remaining if a group of persons at that age were to experience the mortality rates for a particular year over the course of their remaining life. |
|  | Healthy Life Years (HLY) * | Total population, at birth and at age 65 disaggregated by sex. The indicator of healthy life years (HLY) measures the number of remaining years that a person of specific age is expected to live without any severe or moderate health problems. The notion of health problem for Eurostat's HLY is reflecting a disability dimension and is based on a self-perceived question which aims to measure the extent of any limitations, for at least six months, because of a health problem that may have affected respondents as regards activities they usually do (the so-called GALI - Global Activity Limitation Instrument foreseen in the annual EU-SILC survey). The indicator is therefor also called disability-free life expectancy (DFLE). So, HLY is a composite indicator that combines mortality data with health status data. |
|  | Avoidable mortality rate: amenable and preventable deaths | Avoidable mortality: A death is considered avoidable if, in the light of medical knowledge and technology or in the light of understanding of the determinants of health at the time of death, all or most deaths from that cause (subject to age limits if appropriate) could be avoided through good quality healthcare (amenable mortality) or by public health interventions in the broadest sense (preventable mortality). More precisely:  A death is amenable if, in the light of medical knowledge and technology at the time of death, all or most deaths from that cause could be avoided through good quality health care.  A death is preventable if, in the light of understanding of the determinants of health at the time of death, all or most deaths from that cause could be avoided by public health interventions in the broadest sense. |
| **Health Determinants** | Share of population covered by health insurance | Percentage of population covered by i) Government/ social health insurance, ii) private health insurance. |
|  | Life expectancy | Total population, disaggregated by sex and by educational attainment.  Life expectancy at birth, ages 1, 15, 45 and 65 years represents the average number of years of life remaining if a group of persons at that age were to experience the mortality rates for a particular year over the course of their remaining life. |
|  | Body Mass Index * | Disaggregated by age-group, by sex and by educational level (ISCED class 0-2, 3-4, 5-6), by income quintile gap q1/q5.  Proportion of persons who are underweight, normal weight, obese, i.e. whose body mass index (BMI) is <20 kg/m2 for underweight, <25 kg/m2 for normal weight, ≥25 kg/m2 for overweight and ≥30 kg/m2 for obesity. |
|  | Prevalence of different smoking status, self-reported | Diaggregated by sex, age-group (15+, 15-26, 25-64, 65+), educational level (ISCED class 0-2, 3-4, 5-6) and by income quintile gap q1/q5. Proportion of people reporting to i) smoke cigarettes daily, ii) be ex-smokers, iii) have never smoked. |
|  | Opportunities for education: Participation in early childhood education | Enrolment rate in ISCED 0-1 for 4 years old pupils |
|  | Overall experience of life: Life satisfaction | Disaggregated by sex, age, income quintile.  Subjective well-being encompasses three distinct but complementary sub-dimensions: life satisfaction, based on an overall cognitive assessment; affects, or the presence of positive feelings and absence of negative feelings; and eudaimonics, the feeling that one’s life has a meaning. Life satisfaction represents how a person evaluates or appraises his or her life taken as a whole. It is intended to cover a broad, reflective appraisal the person makes of his or her life. The term «life» is intended here as all areas of a person’s existence. The variable therefore refers to the respondent’s opinion/feeling about the degree of satisfaction with his/her life. It focuses on how people are feeling "these days" rather than specifying a longer or shorter time period. The intent is not to obtain the current emotional state of the respondent but to receive a reflective judgement on their level of satisfaction. Satisfaction with particular life domains (financial situation, housing, job, commuting time, living environment, green and recreational areas, time use and personal relationships) also represent a broad subjective assessment of the respetive area taking into account individual situations and preferences. "Meaning of life" represents the eudaimonic aspect of well-being, whether or not the inviduals deem their life as being worthwhile. It is not related to any specific area of life, focuses rather on life in general. The frequency of positive emotions (happiness) in the last four weeks represents the affects dimension of well-being. Trust in others and in institutions (legal system, political system and the police) is of a general nature and it should not apply to a specific group of people or institution. The legal and political system encompass all institutions in the respective categories, whether local, national or transnational. The availability of social support refers to the one's possibility to ask for help (any kind of help: moral, material or financial) from any relatives, friends or neighbours, whether thethe person needs it or not. Only relatives and friends (or neighbours) who don't live in the same household are considered. |
